# Record linkage without patient identifiers: proof of concept using data from South Africa’s national HIV program

**DOI:** 10.21203/rs.3.rs-2893943/v1

**Published:** 2023-05-15

**Authors:** Khumbo Shumba, Jacob Bor, Cornelius Nattey, Dickman Gareta, Evelyn Lauren, William Macleod, Matthew P. Fox, Adrian Puren, Koleka Mlisana, Dorina Onoya

**Affiliations:** University of the Witwatersrand; Boston University; University of the Witwatersrand; Africa Health Research Institute; Boston University; Boston University; Boston University; National Health Laboratory Service; National Health Laboratory Service; University of the Witwatersrand

**Keywords:** Record linkage, HIV, CD4 count, Viral load, South Africa

## Abstract

**Background:**

Linkage between health databases typically requires identifiers such as patient names and personal identification numbers. We developed and validated a record linkage strategy to combine administrative health databases without the use of patient identifiers, with application to South Africa’s public sector HIV treatment program.

**Methods:**

We linked CD4 counts and HIV viral loads from South Africa’s HIV clinical monitoring database (TIER.Net) and the National Health Laboratory Service (NHLS) for patients receiving care between 2015–2019 in Ekurhuleni District (Gauteng Province). We used a combination of variables related to lab results contained in both databases (result value; specimen collection date; facility of collection; patient year and month of birth; and sex). Exact matching linked on exact linking variable values while caliper matching applied exact matching with linkage on approximate test dates (± 5 days). We then developed a sequential linkage approach utilising specimen barcode matching, then exact matching, and lastly caliper matching. Performance measures were sensitivity and positive predictive value (PPV); share of patients linked across databases; and percent increase in data points for each linkage approach.

**Results:**

We attempted to link 2,017,290 lab results from TIER.Net (representing 523,558 unique patients) and 2,414,059 lab results from the NHLS database. Linkage performance was evaluated using specimen barcodes (available for a minority of records in TIER.net) as a “gold standard”. Exact matching achieved a sensitivity of 69.0% and PPV of 95.1%. Caliper-matching achieved a sensitivity of 75.7% and PPV of 94.5%. In sequential linkage, we matched 41.9% of TIER.Net labs by specimen barcodes, 51.3% by exact matching, and 6.8% by caliper matching, for a total of 71.9% of labs matched, with PPV=96.8% and Sensitivity = 85.9%. The sequential approach linked 86.0% of TIER.Net patients with at least one lab result to the NHLS database (N=1,450,087). Linkage to the NHLS Cohort increased the number of laboratory results associated with TIER.Net patients by 62.6%.

**Conclusions:**

Linkage of TIER.Net and NHLS without patient identifiers attained high accuracy and yield without compromising patient privacy. The integrated cohort provides a more complete view of patients’ lab history and could yield more accurate estimates of HIV program indicators.

## BACKGROUND

South Africa’s HIV treatment program is the largest in the world, with about 5.2 million adult patients on antiretroviral therapy (ART) in 2019 (1). Although mass provision of ART has reduced HIV-associated morbidity and mortality (2–6), HIV remains the fifth leading cause of death in South Africa (7) with over 200,000 new infections annually (8).

South Africa’s public sector HIV program is monitored via two administrative databases: the “Three interlinked electronic registers” (TIER.Net) clinical database (9–13) and the National Health Laboratory Service (NHLS) National HIV Cohort (14–23). The NHLS Cohort is the primary laboratory database including all data generated by public-sector medical laboratories while the TIER.Net contains data generated by clinical events at all HIV management centres in South Africa, including ART initiation, ART pick-up dates, regimen type, and clinic visits. Unlike TIER.Net, the NHLS data is nationally deduplicated, enabling longitudinal analyses even when patients re-enter care at other facilities(15, 24). Both national in scope, TIER.Net and NHLS contain complementary data that clinicians draw on for patient care. However, the two databases are not currently integrated, and no consistent patient identifier exists to enable patient-level longitudinal analyses using information from both data sources.

Linkage of health databases traditionally requires primary personal identifiers such as names, national identification numbers, addresses, and phone numbers. However, with heightened concern for data privacy in South Africa and elsewhere, access to patient-identifying information is increasingly restricted to clinical management purposes (25, 26). Validated techniques are therefore required to link databases without primary patient identifiers (27–29) to enable program monitoring, evaluation, and research. A variety of privacy-preserving record linkage (PPRL) approaches have been proposed, with most involving the encryption of primary identifiers behind data owners’ firewalls and linkage of those encoded data (30). Here, we explore the feasibility of linkage without primary patient identifiers at all, relying instead on laboratory event information recorded in both databases. Our paper builds on prior efforts by Edward Nicol et al (31) and Ingrid Basset et al. (32) to link the NHLS with HIV patient management systems for specific clinical cohorts in South Africa.

In this paper, we set out to develop and validate a linkage strategy for the link TIER.Net and the NHLS Cohort without patient identifiers. As a proof of concept, we used data from Ekurhuleni district, a large, mostly urban district in Gauteng province, South Africa. We developed multiple linkage approaches, validated their performance against “gold standard” data, and quantified the benefits of linkage with respect to the completeness of the resulting database. Our goal was to create an integrated HIV cohort with comprehensive clinical and laboratory data that would enable longitudinal analyses of the full HIV care cascade not possible with NHLS or TIER.Net data alone.

## METHODS

### Data and study population

The study population was all patients receiving HIV care in Ekurhuleni District from 1 Jan 2015–31 Dec 2019 at 102 public-sector health facilities with at least one CD4 count or HIV viral load during this period. We compiled data on this study population from two sources: TIER.Net and NHLS.

### Three interlinked Electronic Registers (TIER.Net)

TIER.Net is South Africa’s facility-based electronic patient health data management system. Established in 2010 and scaled up in the following years, TIER.Net serves as the primary monitoring platform for the national HIV care and treatment program (9). Data from patient charts are captured into TIER.Net by clinic staff. TIER.Net contains data on clinical events including ART initiation, ART pick-up dates, regimen type, and clinic visits. While laboratory tests (CD4 counts and HIV viral loads) information are also captured, the process is manual and inconsistent resulting in incomplete f (13). TIER.Net is not nationally networked (9), and lab results preceding HIV diagnosis and ART initiation are largely unavailable on TIER.Net (33, 34). The TIER.Net patient ID is allocated by facilities, and patients who seek care at alternative facilities may receive a new TIER.Net patient ID, creating duplicate records and hindering tracking of patients across facilities (13).

### National Health Laboratory Service (NHLS) National HIV Cohort

The NHLS provides all laboratory and pathology services for the country’s public sector HIV care and treatment program (35). The NHLS maintains a centralised database of all laboratory test data (including CD4 count and HIV viral load (VL) data), with results logged to the NHLS Corporate Data Warehouse (CDW). The NHLS’s CDW previously developed a linkage algorithm. More recently, a team at NHLS, University of Witwatersrand, and Boston University developed, implemented, and validated an improved record-linkage algorithm with much higher sensitivity, enabling analysis of the NHLS database as a national cohort covering all lab-monitored patients in South Africa’s public sector HIV program (14, 15). The NHLS National HIV Cohort has been used to track trends in CD4 counts at presentation, assess retention in care regardless of patient transfer, quantify treatment outcomes for different groups, and evaluate the impact of HIV policy changes (15–22).

### Variables used for linkage Demographic and laboratory test variables

De-identified data were extracted from the TIER.Net and NHLS databases. We extracted laboratory event-specific demographic data [year of birth (YOB), month of birth (MOB), and sex of the patient], geographic data (province name, district name, sub-district name, and health facility name), and details on all CD4 counts and HIV viral loads (result value, test date, and test type) taken between January 1, 2015 and December 31, 2019. The details of each of these linking variables are provided in Box 1. All variables were harmonised between the databases to ensure equivalent formatting. We linked health facilities starting with a crosswalk provided by the National Institute for Communicable Diseases (NICD) at NHLS. We then manually reviewed facility names within Ekurhuleni District to ensure correspondence. All health facility names were standardised to be the same across the two databases. We also retained de-identified unique patient IDs from each database. From TIER.Net, we extracted the “TIER.Net ID”. From NHLS, we extracted a unique patient ID created by NHLS CDW (henceforth, “NHLS CDW ID”) as well as the National HIV Cohort ID (henceforth, “NHLS Cohort ID”). At the time of writing, the NHLS Cohort ID was available only through March 2018.

### Specimen barcodes as a gold standard matching variable

Some TIER.Net laboratory results were recorded with their NHLS specimen barcode. These alphanumeric barcodes are allocated centrally by NHLS and are not duplicated within or across health facilities. Barcodes are affi xed to biological specimens (e.g. blood test tubes), the corresponding NHLS test request form, the facility’s specimen register, and are provided with the test results sent back to facilities from NHLS. The barcode is the same across all the tests performed on the same biological specimen. Except for cases where the same test was repeated on the same specimen, the combination of barcode and test type is unique. Barcodes are available for nearly all laboratory results in the NHLS database. Although the barcode data are highly incomplete in TIER.Net and cannot be used as the only linkage strategy, the combination of barcode and test type offers a highly accurate “gold standard” for validation of other approaches.

To confirm the suitability of using barcodes as a gold standard, we assessed the concordance of other test information (YOB, MOB, sex, test date and value, and facility name) when barcodes matched. We first excluded labs where the specimen barcodes and test type were not unique (0.013%-TIER.Net and 0.005%-NHLS). We then identified lab results with the same barcode and test type in the two databases and quantified the % discordance in the associated test information. To assess the probability of barcode matching by chance, we randomly selected 100,000 lab results from TIER.net and linked them to 100,000 randomly sampled lab results from NHLS (**Table S1**). The expectation was that a very small share of these randomly selected pairs would be true matches. We quantified the proportion of discordance in the randomly-matched pairs. By comparing the share of barcode matches that were fully discordant in other characteristics with the share that would be expected to be fully discordant by random chance, we were able to estimate the false positivity rate in the barcode matching, under the assumption that all fully discordant records were different people. This is an upper bound on the false positivity rate, given that some of the fully discordant barcode matches actually may have been true matches with a lot of typographic error.

### Exclusions before linkage

**Figure 1:**
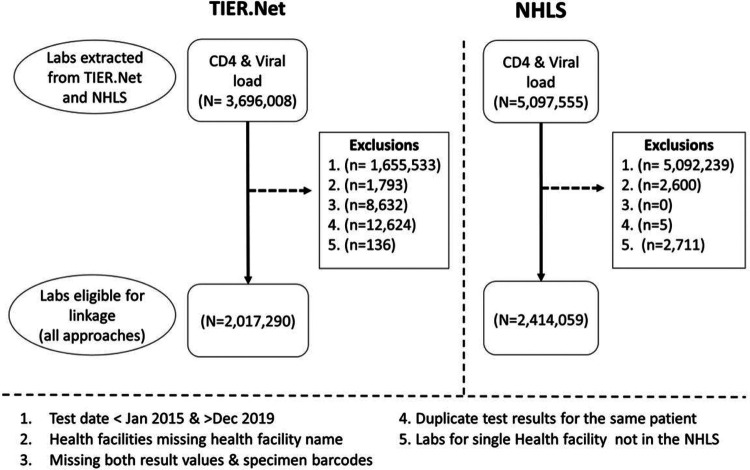
Flow diagram: laboratory results from TIER.Net and NHLS submitted for linkage

At the time of analysis, we had access to TIER.Net and NHLS labs data available from 2004–2020. Our linkage was performed on all CD4 count and viral load tests in TIER.Net and NHLS with a test date between 1st January 2015 and 31st December 2019, subject to the following exclusions (Fig. 1): (1) Health facilities not found in both databases; (2) labs entries with missing result values and duplicate test results per TIER.Net patient. The remaining laboratory results were considered “eligible labs” for inclusion in the analysis. Stata v16.1 was used in all data processing and analyses.

### Methods for Record Linkage

We applied four record linkage approaches using the laboratory test result information.

- Barcode matching: Matching based on the specimen barcode and test type.
- Exact matching. Matching on exact values of the linkage variables: YOB, MOB, facility name, sex, test date, test type, and test result value (LDL viral loads were coded as 0 in both datasets).
- Caliper matching. Matching using exact values of sex, facility name, test type, and test value while allowing for approximate matches on test date (± 5 days). Viral load test results can be denoted as being “lower than detectable” (LDL) values based on the sensitivity cut-offs of the testing methods, which may differ across laboratories and over time (36). Since multiple patients may have VLs under LDL for tests requested on the same date and in the same facility, these labs may not be considered unique for linkage. We, therefore, coded all viral load “LDL” entries as “0” for linkage and validated the linking of these labs by testing the uniqueness of variable combinations (sex, test date, test facility, YOB and MOB) excluding the result value.
- Sequential linkage. Finally, we implemented the three methods above sequentially, using the most accurate method available for each record pair to maximise yield while maintaining high accuracy (matching by specimen barcodes where available, then the exact matching strategy and lastly caliper-matching for the remaining unmatched data).

### Evaluating performance of the linkage methods

We used the subset of laboratory tests with specimen barcodes in TIER.Net and NHLS to assess the performance of the exact and calliper matching linkage strategies. We assumed that barcodes were missing completely at random. Record pairs where the specimen barcode matched were defined as “true matches”. Record pairs where the specimen barcode did not match were defined as “true non-matches”. Performance was assessed across four dimensions: sensitivity, positive predictive value (PPV), linkage yield and enrichment of the TIER.Net laboratory profile because of the linkage. Definitions are provided below:

- Sensitivity. We computed the sensitivity as the proportion of “true matches” (i.e. barcode matches) that were identified by each linkage approach.
- Positive predictive value (PPV). We computed PPV as the proportion of matches identified by each linkage approach that were a “true match”.
  The approach for estimation of these parameters for the sequential linkage is provided in **Text S1**. For sensitivity and PPV, we estimated exact binomial 95% confidence intervals using the one-sample Clopper–Pearson method (37). Because there are a very large number of true non-matches, specificity and negative predictive value are nearly 100% and are not reported (21).
- Linkage yield. We defined linkage yield at both the laboratory test result and patient levels for tests conducted between 2015 and 2019. We defined test-level yield as the proportion of TIER.Net laboratory results that could be linked to NHLS. We defined patient-level yield as the proportion of TIER.Net patients with laboratory results that were linked to NHLS based on at least one test result. We note that yield is distinct from sensitivity, which uses as a denominator the share of true matches between the datasets.
- Enrichment. The NHLS National HIV Cohort deduplicated ID was available from January 2004 to March 2018. For each linkage strategy, we identified and extracted all laboratory results in the NHLS National HIV Cohort (Jan 2015 – Dec 2018) that were associated with that TIER.Net-linked NHLS Cohort ID. This linkage enriched the TIER.Net data with additional laboratory results not existing in TIER.Net, creating a more comprehensive profile of CD4 counts and HIV viral loads for patients in TIER.Net. We defined enrichment of the TIER.Net database at the % increase in the number of CD4 counts and viral loads) associated with patients in TIER.Net after the linkage with the NHLS National HIV Cohort, relative to the number of CD4 counts and viral loads in TIER.Net alone.

Linkage yield and enrichment were calculated on the complete sample of TIER.Net records with laboratory results included in the linkage exercise.

## RESULTS

### Description of the data used for linkage

After implementing the exclusion criteria to the 3,696,008 TIER.Net labs from 2004–2020, a total of 2,017,290 “eligible TIER.Net labs” (668,900 CD4 counts; 1,348,390 viral loads) for 523,558 patients in TIER.Net were available for linkage, of which 605,506 (30.0%) were LDL viral loads. Figure 1 presents the sample selection flow diagram. After the same exclusions were applied to 5,097,555 NHLS labs from 2004–2020, a total of 2,414,059 “Eligible NHLS labs” (CD4 = 836,633; VL = 1,577,426) were available for linkage of which 688,277 (28.5%) were LDL viral loads. Overall, 684,973 (34.0%) eligible TIER.Net labs had specimen barcodes, compared to 2,410,230 (99.8%) eligible NHLS labs (Table 1). **Figure S1** shows the number of lab results in TIER.Net and NHLS in Ekurhuleni District over time. NHLS had more lab results than TIER.Net throughout the study period, with about 25% more than TIER.Net in 2015 and about 15% more in 2019.

### Validation of the barcode as a suitable gold standard

Linking the TIER.Net and NHLS data using only the specimen barcode linked 608,210 (88.8%) of TIER.Net lab data that had a barcode. Among the lab results with matching barcodes and test types, only 4.3% were discordant across more than one other characteristic (YOB, MOB, sex, test date and value, and facility name), and just 0.03% were discordant across all six characteristics. To assess the probability of discordant characteristics occurring by chance, we randomly matched 100,000 lab results from TIER.net to 100,000 lab results from NHLS. Among these random pairs, 99.9% were discordant on two or more characteristics and 52.7% were discordant on at least five characteristics. Under the assumption that all fully discordant pairs were different people (even if there was a barcode match), we estimated that 0.06% (0.03/52.7) of all barcode matches were false positives. This investigation revealed that the combination of barcode and test type had greater than 99.9% PPV, making it a suitable gold standard for validation of other approaches.

### Linkage approaches that uniquely identify laboratory results within each database

To identify potentially effective linking strategies, we assessed what share of lab results were uniquely identified in the TIER. The combination of test type, test date, facility, sex, YOB and MOB uniquely identified a higher proportion of labs in both TIER.Net and NHLS (TIER.Net = 99.0%, NHLS = 99.1%) compared to replacing YOB and MOB with age at test (TIER.Net = 92.1%, NHLS = 91.6%). This difference was particularly important for the linkage of LDL viral loads, which lacked discriminating information in the result value field (Table 2). These investigations guided our selection of variables for exact and caliper matching.

#### Sensitivity and positive predictive value for exact and caliper matching vis-à-vis barcode “gold standard” matching using NHLS-TIER.Net validation data

We evaluated exact and caliper matching using barcodes as a gold standard. We identified 608,210 lab records with identical barcodes in TIER.Net and NHLS and considered these to be “true matches”. All other pairs of laboratory records from TIER.Net and NHLS in which barcodes differed (and were non-missing) were considered “true non-matches”. Exact matching yielded a total of 441,300 matches, of which 419,658 were “true matches”, a sensitivity of 69.0% (95%CI: 68.9, 69.1) and a PPV of 95.1% (95%CI: 95.0, 95.2). Caliper matching yielded a total of 487,077 matches, with a sensitivity of 75.7% (95% CI: 75.6, 75.8) and a PPV of 94.5% (95%CI: 94.4, 94.6).

### Linkage performance and yield in the complete sample

**Figure 2:**
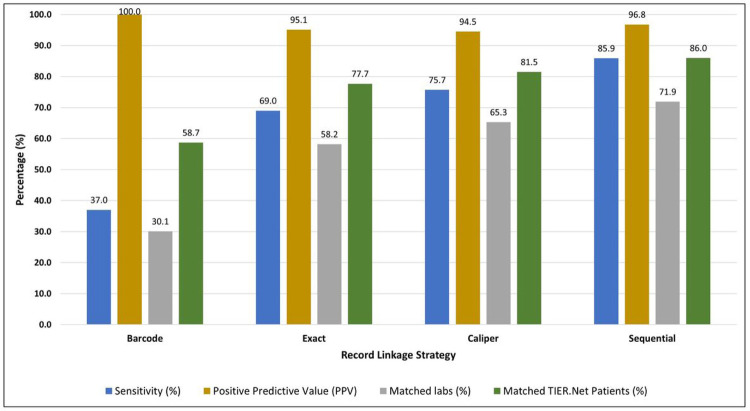
Record linkage performance for each linkage strategy in the full dataset Note: The figure compares the estimated performance of each linkage strategy with respect to sensitivity, positive predictive value (PPV), lab-level linkage yield, and patient-level linkage yield. Estimates were based on extrapolation from the barcode subsample, under the assumption that barcodes were missing completely at random (S1 Text). Overall, the sequential linkage approach outperformed the other approaches with the highest linkage yield at the lab (71.9%) and patient level (86.0%), high sensitivity (85.9%), and PPV (96.8%).

Using our calculations of sensitivity and PPV from the “gold standard” dataset, we then estimated these parameters for the complete sample of eligible TIER.Net and NHLS labs data when linked using four methods: barcodes matching, exact matching, caliper matching, and sequential linkage (Fig. 2; **Table S2**). We additionally assessed “yield”, i.e. the proportion of lab results and patients in TIER.Net that were linked to NHLS by each method.

Of all eligible TIER.Net labs, 608,210 labs matched on barcodes. Since all barcode matches were gold standard matches, PPV of this strategy was 100%, but sensitivity was estimated at just 95.8% and 59% of TIER.Net patients were matched. Exact matching linked 1,174,983 lab results; 41% of TIER.Net labs and 78% of TIER.Net patients were linked using this approach. Caliper matching had a greater yield with 1,317,429 matches (65% of all TIER.Net labs and 81% of TIER.Net patients with labs captured between 2015 and 2019).

The sequential linkage approach combined all three strategies to maximise yield. Among the eligible labs, the sequential linkage matched 1,450,787 records, of which 608,210 were matched by barcode, 743,993 were exact matched, and 98,584 were caliper matched (and were not exact matches). This approach yielded the highest linkage yield, at 71.9% of TIER.Net labs matched. Fully, 86% of all TIER.Net patients with a CD4 count or viral load could be matched to the NHLS database using this approach. We estimated a sensitivity of 85.9%. We estimated PPV at 96.8%, implying that just 3.2% of matches were false positives (Fig. 2; **Table S2**).

### Enrichment of TIER.Net through linkage to the NHLS National HIV Cohort

**Figure 3:**
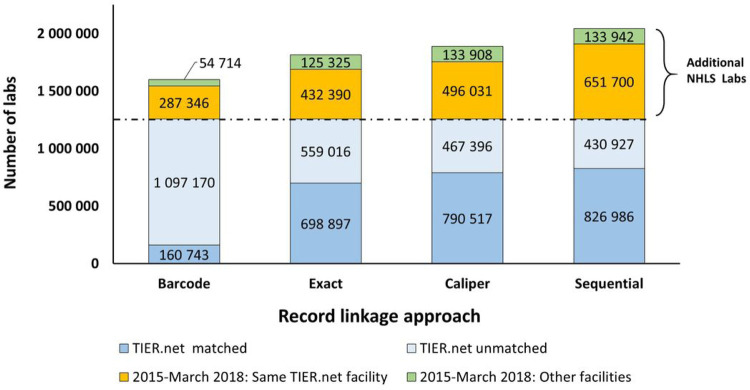
Enrichment of lab results in TIER.Net through linkage with NHLS National HIV Cohort (lab data generated between January 2015-March 2018)

At the time of writing, the NHLS National HIV Cohort ID was available for laboratory tests conducted between January 2004 and March 2018 (Table 1). The total number of additional labs identified for the period spanning Jan 2015-March 2018 from the NHLS National HIV Cohort for each strategy varied. Barcode matching led to a 27.2% increase (n = 342,060) in lab results relative to TIER.Net alone. Exact matching led to a 44.6% increase (n = 560,715). Caliper matching led to a 50.1% increase (n = 629,939). And sequential linkage led to a 62.5% increase in the number of CD4 counts and viral loads relative to TIER.Net alone (n = 785,642). These numbers mean that before the linkage to the NHLS National HIV Cohort, TIER.Net contained only 61.6% of all available CD4 counts and viral loads conducted between January 2015-March 2018. Of the 785,642 CD4 counts, and viral loads newly identified through linkage to the NHLS National HIV Cohort, 133,942 (17.0%) were conducted at different facilities than the linking lab event from the TIER.Net facility (Fig. 3).

## DISCUSSION

We developed and validated an algorithm to link two large administrative health databases – TIER.Net and the NHLS National HIV Cohort – without patient identifiers. The choice of record linkage methods depends on the objectives of the study (28, 29). Our goal was to leverage the comprehensive laboratory data available in the NHLS National HIV Cohort to complete the laboratory test profile for HIV patients observed in TIER.Net using privacy-preserving linkage methods. South African studies have linked the NHLS data with other patient registries before the enactment of the POPIA (38–42). However, while some studies have attempted to link patient management systems in South Africa without patient identifiers (31, 32), none have done so using large-scale data. We developed a simple linkage strategy using information on laboratory tests recorded in both NHLS and TIER.Net, without accessing primary patient identifiers such as names or national ID numbers.

We established that a combination of individually non-identifying variables including basic demographic fields (year/month of birth and sex), health facility, and laboratory result (test type, test date, and test value) can be used to link the TIER.Net and NHLS databases with high accuracy and substantial linkage yield. A sequential record linkage strategy, leveraging barcodes for about a quarter of the data, linked 86% of TIER.Net patients to the NHLS database with a Type 1 error rate of less than 4%. Combining TIER.Net with the NHLS National HIV Cohort increased the number of laboratory results of patients in TIER.Net by 62.5% relative to TIER.Net alone, indicating that the NHLS Cohort fills significant gaps in patient information in TIER.Net. Because our approach protects patient confidentiality and privacy, it may be generalizable in contexts where access to primary patient identifiers is limited.

This study has several limitations. First, the accuracy of test dates and test value data in TIER.Net are unclear, limiting the application of our linkage strategy for locations and periods before 2015 with considerable missing values and data errors. Second, several facilities could not be linked between TIER.Net and NHLS and were excluded, highlighting the need for a national, regularly maintained crosswalk with NHLS and Department of Health facility identifiers. Third, the NHLS National HIV Cohort was created using a validated algorithm, and like all deduplication efforts contains some matching errors. Fourth, not all laboratory results in TIER.Net could be linked to NHLS. We were unable to accurately link 28% of CD4 count and viral load results in TIER.Net to NHLS. We cannot be sure why they were not linked; however, other studies have noted inconsistencies between information recorded in patient files and that captured in TIER.Net (12, 13, 34, 44). Fifth, our approach requires the availability of data on the same patient characteristics – here, laboratory test results – to facilitate linkage, and would not be suitable for linking databases that do not contain some shared data points. Finally, the study was limited to one mostly urban district in South Africa, although the methods are likely generalizable more broadly.

## CONCLUSION

Despite the exploratory nature of our study, the findings offer an exciting and readily available template for rapid integration of the NHLS National HIV Cohort and TIER.Net patient management system without compromising patient privacy and confidentiality for HIV research and policy evaluation in South Africa. Because 14% of TIER.Net patients with laboratory results – and all TIER.Net patients without laboratory results – remained unlinked, other methods, including the use of patient identifiers, should be used to create a comprehensive database for patient care and monitoring purposes.

## Figures and Tables

**Table 1:** Description of TIER.Net and NHLS datasets used in the linkage

Parameter	TIER.Net	NHLS
*January 2015 - December 2019*		
Total number of lab results #	2017290	2414059
Viral loads # (%)	1348390 (66.8%)	1577426 (65.3%)
CD4 counts # (%)	668900 (33.2%)	836633 (34.7%)
Lab results with barcodes # (%)	684973 (34.0%)	2410230 (99.8%)
Number of patients with lab results # (ID)	523558 (TIER_ID)	985734 (CDW_ID)
*January 2015 - March 2018*		
Total number of lab results #	1257913	1545467
Number of patients with lab results # (ID)	403476 (TIER_ID)	703231 (CDW_ID) 479883 (Cohort_ID)

Note: The table provides a summary of the NHLS and TIER.Net data used in the record linkage. Data are for patients receiving care at 102 facilities in Ekurhuleni province, January 2015 – December 2019. TIER_ID = unique patient identifier in TIER.Net; CDW_ID = unique patient identifier created by the NHLS Corporate Data Warehouse; Cohort_ ID = improved unique patient identifier created for the NHLS National HIV Cohort.

**Table 2:** Percent of lab results that are uniquely identified based on the following characteristics.

	Within TIER.Net		Within NHLS	
	All labs	LDL VL only	All labs	LDL VL only
Number of lab results	2017290	605506	2414059	688277
*Of which, the number of labs (%) results uniquely identified by*:				
(a) Test type, date, facility	435778 (89.3%)	182953 (30.2%)	245790 (10.2%)	115435 (16.8%)
(b) Test type, date, facility, sex, age	1801993 (89.3%)	557568 (92.1%)	2138481 (88.6%)	630540 (91.6%)
(c) Test type, date, facility, sex, YOB, MOB	1989169 (98.6%)	599981 (99.1%)	2376874 (98.5%)	681363 (99.0%)
(d) Test type, date, facility, sex, age, result value	1986155 (98.5%)	577466 (95.4%)	2358088 (97.7%)	632642 (91.9%)
(e) Test type, date, facility, sex, YOB, MOB, result value	2011903 (99.7%)	602435 (99.5%)	2407320 (99.7%)	681607 (99.0%)

Note: YOB = Year of birth; MOB = Month of birth. The table illustrates the different linkage variable combinations considered in our linkage strategies for the entire dataset and lower than detectable (LDL) viral loads.

## Data Availability

The de-identified data underlying this article were provided by South Africa’s National Department of Health and the NHLS. For more information on the NHLS National HIV Cohort, we refer the reader to MacLeod et al. 2022 (15). Data and analysis scripts will be shared on request to the corresponding author with permission from the two parties.

## Key Message

- Clinical data linkage without identifiers is a useful tool for researchers and policymakers to analyze health-related data while protecting patient information.
- We linked data from South Africa’s TIER.Net clinical database and the National Health Laboratory Service (NHLS) National HIV Cohort without patient identifiers, protecting patient confidentiality.
- A sequential approach – matching on barcode (where available), laboratory result value, specimen collection date, facility of collection, patient year and month of birth, and sex – linked 86% of TIER.Net patients with the NHLS data with a 3.2% type I error rate (1 – PPV).
- Integrating TIER.Net with the NHLS National HIV Cohort increased the number of HIV monitoring laboratory results by 62.6% relative to TIER.Net alone.
- Piloted in Ekurhuleni, a large urban district, this linkage approach offers novel opportunities to evaluate South Africa’s public sector HIV program using an integrated NHLS-TIER.Net HIV cohort.

## Patient and Public Involvement

The study design was informed by past interactions with patients and healthcare providers, but patients and/or the general public were not directly involved in the research’s design, implementation, or reporting.

## Box 1. Description of the linkage variables

The following variables were used to link laboratory tests in TIER.Net and the NHLS National HIV Cohort:

- *Specimen Barcode –* Each blood specimen is assigned a unique NHLS specimen barcode.
- *Test type* – CD4 count or HIV viral load.
- *Test facility –* Facility where a specimen was taken (NHLS) or recorded (TIER.Net).
- *Test taken date –* “Taken date” in NHLS and “result date” in TIER.Net
- *Test result value –* Numeric value of CD4 count or viral load test result. Some viral loads were classified as “lower than detectable limit”; these were coded as “0” for linkage.
- *Year and month of birth –* extracted from the date of birth recorded for each NHLS specimen and each TIER.Net patient record.
- *Biological Sex –* recorded in TIER.Net and NHLS as “Male” or “Female”

## Abbreviations

AARMS: Academic Affairs and Research Management System
ART: Antiretroviral therapy
CDW: Corporate Data Warehouse
CI: Confidence Interval
HIV: Human immunodeficiency virus
HREC: Human Research Ethics Committee
ID: Identification Number
LDL: Lower than detectable
MOB: Month of birth
NHLS: The National Health Laboratory Service
NICD: National Institute for Communicable Diseases
POPI: Protection of Personal Information
PPRL: privacy-preserving record linkage
PPV: Positive Predictive Value
TIER: Net Three interlinked Electronic Registers
YOB: Year of birth
